# Antiprotozoal Activity of Thymoquinone (2-Isopropyl-5-methyl-1,4-benzoquinone) for the Treatment of *Leishmania major*-Induced Leishmaniasis: In Silico and In Vitro Studies

**DOI:** 10.3390/antibiotics11091206

**Published:** 2022-09-06

**Authors:** Kamal A. Qureshi, Mahrukh Imtiaz, Ibrahim Al Nasr, Waleed S. Koko, Tariq A. Khan, Mariusz Jaremko, Syed Mahmood, M. Qaiser Fatmi

**Affiliations:** 1 Department of Pharmaceutics, Unaizah College of Pharmacy, Qassim University, Unaizah 51911, Saudi Arabia; 2 Department of Biosciences, COMSATS University Islamabad, Islamabad 45600, Pakistan; 3 Department of Laboratory Sciences, College of Science and Arts, Qassim University, Ar Rass 51921, Saudi Arabia; 4 Department of Biology, College of Science and Arts, Qassim University, Unaizah 51911, Saudi Arabia; 5 Department of Clinical Nutrition, College of Applied Health Sciences, Qassim University, Ar Rass 51921, Saudi Arabia; 6 Smart-Health Initiative (SHI) and Red Sea Research Center (RSRC), Division of Biological and Environmental Sciences and Engineering (BESE), King Abdullah University of Science and Technology (KAUST), Thuwal 23955, Saudi Arabia; 7 Department of Pharmaceutical Technology, Faculty of Pharmacy, Universiti Malaya, Kuala Lumpur 50603, Malaysia

**Keywords:** antileishmanial activity, molecular docking, molecular dynamics simulation, thymoquinone, squalene monooxygenase

## Abstract

Leishmaniasis, a neglected tropical parasitic disease (NTPD), is caused by various *Leishmania* species. It transmits through the bites of the sandfly. The parasite is evolving resistance to commonly prescribed antileishmanial drugs; thus, there is an urgent need to discover novel antileishmanial drugs to combat drug-resistant leishmaniasis. Thymoquinone (2-isopropyl-5-methyl-1,4-benzoquinone; TQ), a primary pharmacologically active ingredient of *Nigella sativa* (black seed) essential oil, has been reported to possess significant antiparasitic activity. Therefore, the present study was designed to investigate the in vitro and in silico antileishmanial activity of TQ against various infectious stages of *Leishmania major* (*L. major*), i.e., promastigotes and amastigotes, and its cytotoxicity against mice macrophages. In silico molecular dockings of TQ were also performed with multiple selected target proteins of *L. major*, and the most preferred antileishmanial drug target protein was subjected to in silico molecular dynamics (MD) simulation. The in vitro antileishmanial activity of TQ revealed that the half-maximal effective concentration (EC_50_), half-maximal cytotoxic concentration (CC_50_), and selectivity index (SI) values for promastigotes are 2.62 ± 0.12 μM, 29.54 ± 0.07 μM, and 11.27, while for the amastigotes, they are 17.52 ± 0.15 μM, 29.54 ± 0.07 μM, and 1.69, respectively. The molecular docking studies revealed that squalene monooxygenase is the most preferred antileishmanial drug target protein for TQ, whereas triosephosphate isomerase is the least preferred. The MD simulation revealed that TQ remained stable in the binding pocket throughout the simulation. Additionally, the binding energy calculations using Molecular Mechanics Generalized-Born Surface Area (MMGBSA) indicated that TQ is a moderate binder. Thus, the current study shows that TQ is a promising antileishmanial drug candidate that could be used to treat existing drug-resistant leishmaniasis.

## 1. Introduction

Leishmaniasis is a neglected tropical parasitic disease (NTPD) disseminated by female phlebotomine sandflies. It is caused by various species of obligate intracellular *Leishmania* protozoan parasites [1,2,3,4,5]. In humans, the parasite grows and reproduces as an internal amastigote inside macrophage phagolysosomes [3]. Three types of leishmaniasis have been described: cutaneous leishmaniasis, the most prevalent type, caused by *L. donovani*, *L. tropica*, and *L. aethiopica*; mucocutaneous leishmaniasis, caused by *L. braziliensis*; and visceral leishmaniasis, commonly known as Kala-azar, induced by *L. donovani* [6]. Visceral leishmaniasis is the most severe type [3]. At present, there is no available vaccine for human leishmaniasis; thus, the only treatment option is chemotherapy [1].

There are three frequently recommended antileishmanial medications, i.e., amphotericin B, paromomycin, and miltefosine; however, their use is limited due to cytotoxicity or very high cost. Thus, the current therapeutic repertoire is limited, and the development of resistance to such medications is a major concern, particularly in Saharan and sub-Saharan countries [1]. The effective control of drug-resistant leishmaniasis requires the urgent development of novel drugs and the identification of new targets.

Over the past decade, financing for antiparasitic drug discovery has shifted dramatically. Numerous organizations, including the Institute of One World Health (IOWH), the Drugs for Neglected Diseases Initiative (DNDi), and the Bill and Melinda Gates Foundation, have contributed financially to the development of drugs for tropical diseases by promoting scientific discoveries in the industry and in academia, including publicly available genomic sequencing, which aided enormously in the drug discovery and development processes [1]. The availability of whole-genome sequencing data of several *Leishmania* species, including *L. major*, *L. braziliensis*, and *L. infantum*, has significantly improved drug research and development [7].

**Figure 1:**
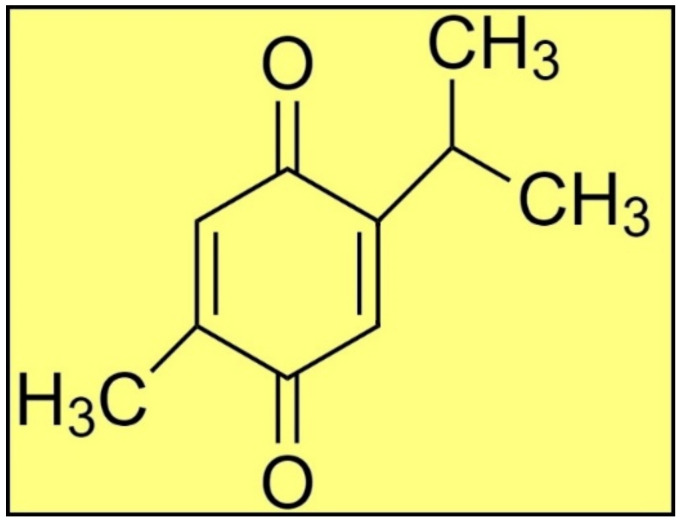
The structure of TQ.

Plants produce a range of secondary metabolites to combat various pathogens in their surroundings [8,9,10,11,12,13]. For example, *Nigella sativa (N. sativa)* is an invaluable plant that grows naturally in southern Europe, northern Africa, and southern Asia. Historically, this plant’s seeds and leaves are employed as a flavoring agent in Asian and Middle Eastern cuisines [14]. TQ (Figure 1) is a main bioactive phytoconstituent of *N. sativa* essential oil, and exhibits several pharmacological properties, e.g., antimicrobial, antiparasitic, antiviral, anti-inflammatory, anticancer, etc. [14,15,16,17,18,19]. Thus, the present study was designed to investigate the antiprotozoal activity of TQ against *L. major* induced-leishmaniasis through in silico and in vitro studies. The in vitro antileishmanial activity of TQ was evaluated against the promastigote and amastigote infectious stages of *L. major.* The in silico molecular docking studies were performed between TQ and 16 previously reported antileishmanial target proteins [5] through the detailed analyses of the intermolecular interactions between the targets and TQ. The MD simulation was performed between TQ and the most preferred antileishmanial target protein along with MMPBSA binding energy evaluations.

## 2. Results

Fourier transform infrared spectroscopy (FT-IR) revealed that the procured TQ had the same spectroscopic spectrum as the standard TQ (Figures S1 and S2, see Supplementary Materials) [5].

### 2.1. In Vitro Antileishmanial Activity of TQ

#### 2.1.1. Antipromastigote Activity

**Figure 2:**
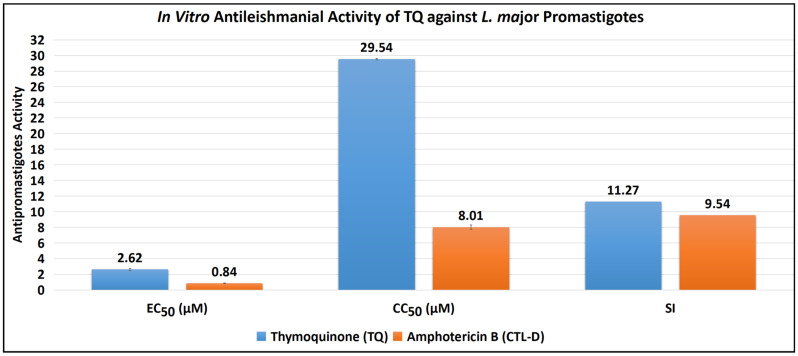
Antipromastigote activity of TQ and CTL-D. Note: All the tests were conducted in triplicate, and the results are presented as mean ± SD.

The antipromastigote assay revealed that TQ has substantial antipromastigote activity, as shown by EC_50_ values of 2.62 ± 0.12 μM, CC_50_ values of 29.54 ± 0.07 μM, and SI values of 11.27. The EC_50_, CC_50_, and SI values for the control drug (CTL-D), i.e., amphotericin B, were 0.84 ± 0.04 μM, 8.01 ± 0.29 μM, and 9.54, respectively (Table 1 and Figure 2).

#### 2.1.2. Antiamastigote Activity

**Figure 3:**
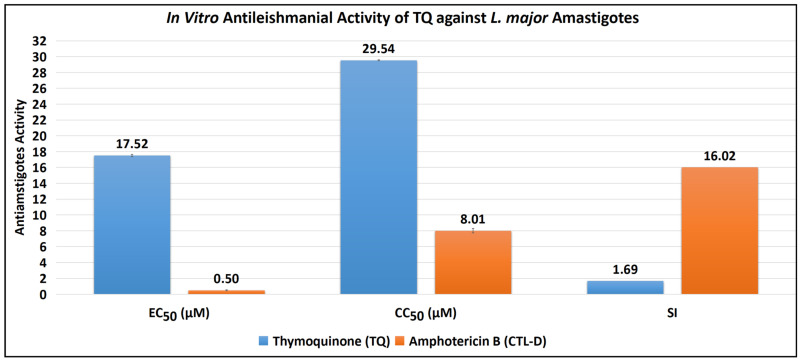
Antiamastigote activity of TQ and CTL-D.

**Figure 4:**
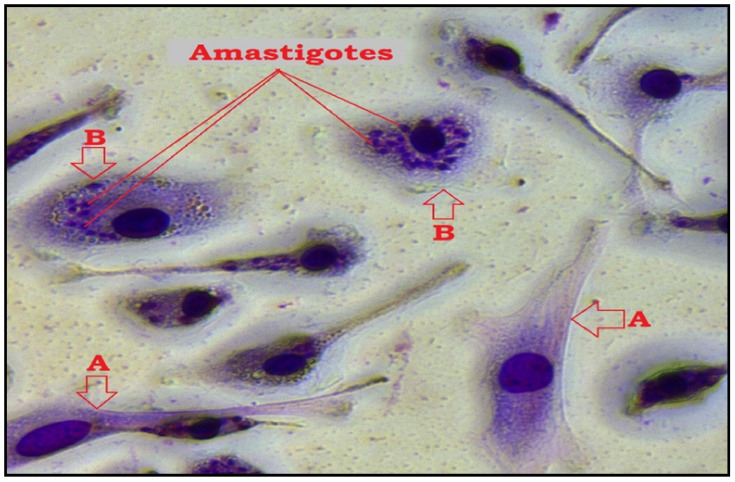
Microscopic image of macrophage infection by *L. major* after TQ treatment and Giemsa staining using 40× objective (Leica MCD-400). (**A**); uninfected macrophage, (**B**); infected macrophage while amastigotes appear as darkly stained blue particles in the cytoplasm.

The antiamastigote assay revealed that TQ has substantial antiamastigote activity, as shown by EC_50_ values of 17.52 ± 0.15 μM, CC_50_ values of 29.54 ± 0.07 μM, and SI values of 1.69. The EC_50_, CC_50_, and SI values for the control drug (CTL-D), i.e., amphotericin B, were 0.50 ± 0.06 μM, 8.01 ± 0.29 μM, and 16.02, respectively (Table 2, Figure 3 and Figure 4).

### 2.2. In Silico Antileishmanial Activity of TQ

#### 2.2.1. Molecular Docking Studies

Sixteen reported antileishmanial enzymes [5] were selected to dock with TQ, and the details of the docking results are presented in Table 3, which indicate that squalene monooxygenase exhibits the best interactions with TQ.

**Figure 5:**
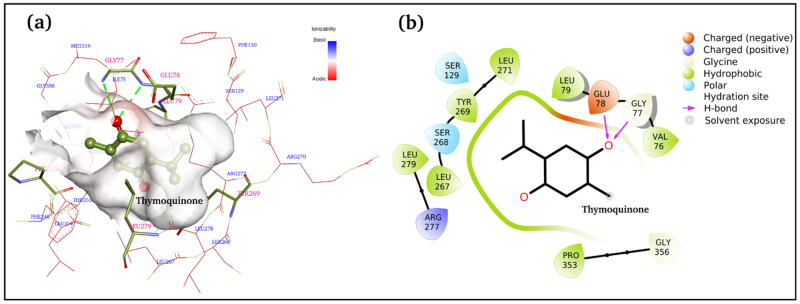
Docked complex of TQ and squalene monooxygenase with details of the binding interactions displayed. (**a**) 3D plot and (**b)** 2D plot.

Figure 5a,b displays 3D and 2D binding site interactions for TQ-squalene monooxygenase complex. As observed in the molecular docking study, Glu78 and Gly77 are directly involved in H-bond formation with TQ. The hydrophobic interactions are more prominent, and residues Val76, Leu79, Leu267, TYR269, Leu271, Leu279, and Pro353 are involved in hydrophobic interactions with the TQ. Arg277 is electrostatically interacting with the carbonyl group. The overall complex is fairly stable, with a −7.1 kcal/mol binding score. To study the structure and dynamics, this complex was subjected to MD simulation along with the reference control, i.e., the TQ-free protein.

#### 2.2.2. MD Simulations

**Figure 6:**
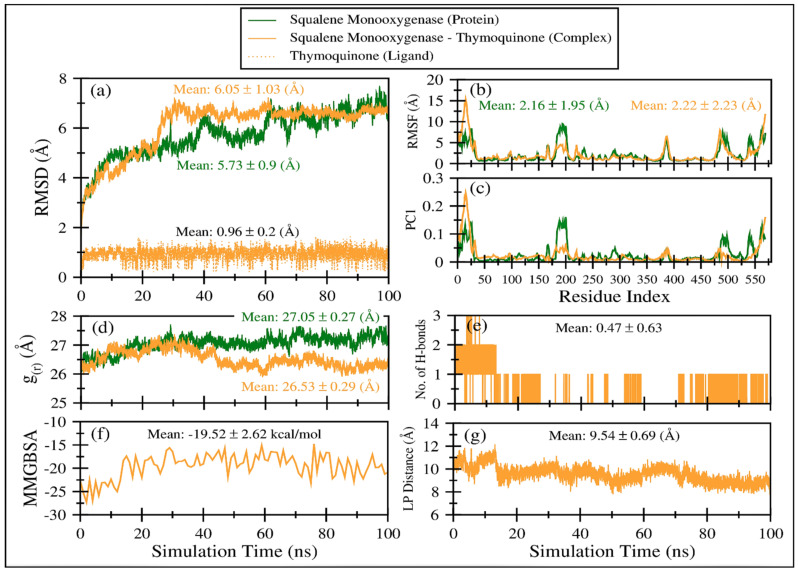
Various comparative analyses were performed on the trajectories of squalene monooxygenase in TQ-free (green line) and bound states (solid orange line). (**a**) Root mean square deviations using Cα atoms of the protein. (**b**) Root mean square fluctuations using Cα atoms of the protein. (**c**) Fluctuations as obtained from the principal analysis 1 using Cα atoms of the protein. (**d**) Radius of gyration based on Cα atoms of the protein. (**e**) Number of hydrogen bonds between TQ and squalene monooxygenase. (**f**) TQ and squalene monooxygenase binding energy as obtained from MMGBSA calculations. (**g**) Center-of-mass distance between TQ and squalene monooxygenase during the course of the MD simulation.

Figure 6a displays the comparative root mean square deviation (RMSD) of squalene monooxygenase in TQ-free (green line) and bound states (solid orange line). It is apparent that both systems exhibit slightly higher RMSD values (i.e., >5.7 Å), indicating that the proteins have undergone conformational changes with respect to their initial structures. Overall, both protein systems show a negligible difference in mean RMSD values; TQ-bound squalene monooxygenase exhibits a more stable RMSD plot after 30 ns. On the other hand, the RMSD curve for TQ-free squalene monooxygenase keeps increasing, although still within the range of ~2 Å. This clearly indicates that TQ induces stability in the protein complex. Figure 5a also plots the RMSD of TQ (dotted orange line) during 100 ns of simulation time, with a mean value of 0.96 ± 0.2 Å. Negligible fluctuations are observable; however, the overall RMSD curve remains very stable throughout the simulation, indicating that there is a good balance between the flexibility and rigidity of TQ within the binding pocket of the protein.

Figure 6b displays comparative root mean square fluctuations (RMSF) of squalene monooxygenase protein in TQ-free (green line) and bound states (solid orange line). TQ-induced constraints in atomic fluctuations are prominent in regions 175–205, representing a loop. As the N-terminal region is more flexible in the TQ-bound complex (snapshots are displayed in Figure S3), the overall RMSF value for the TQ-bound complex is negligibly higher (2.22 ± 2.23 Å) than TQ-free squalene monooxygenase protein (2.16 ± 1.95 Å). Figure S3 displays snapshots of protein system at various timescales, which indicate that protein’s scaffold remains stable throughout the simulation time; however, the loops’ conformations are invariably changed. These results align well with the observations in RMSF and principal components analysis.

**Figure 7:**
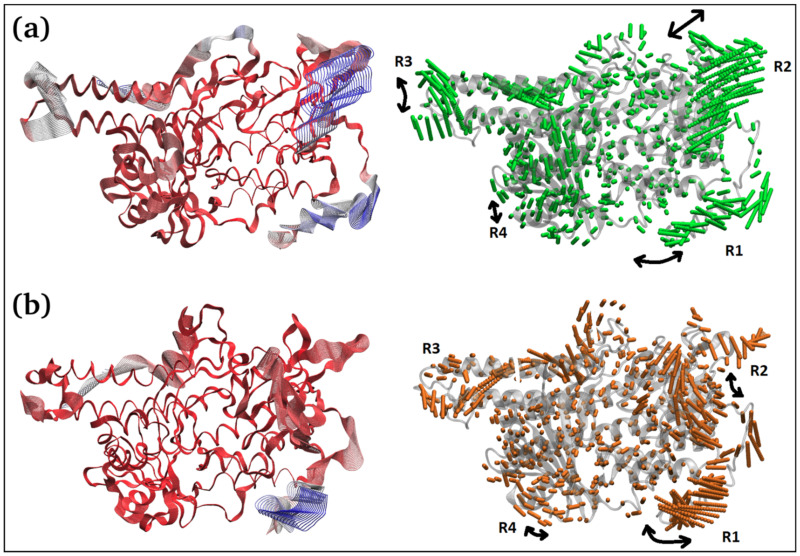
Two different representations of the interpolated structures of the TQ-free (**a**) and bound states (**b**) of the squalene monooxygenase protein complex along PC1 produced by the mktrj.pca() function. On the left, atoms are colored on a scale from blue to red, where blue represents atoms showing large motion amplitudes, and red represents more rigid atoms. Images on the right show the amplitude of motion via a different mode of representation.

The principal component 1 (PC1) analysis (Figure 6c and Figure 7) captures the dominant mode of motion in TQ-free and bound squalene monooxygenase protein. It can be observed that various loop regions are more flexible in TQ-free squalene monooxygenase protein (as indicated by blue colors on left-side images). The right two images show the path in 3D space occupied by the dominant motion of protein during simulation. For ease of understanding, the protein regions are labeled as R1, R2, R3 and R4, and their pattern of motions is represented by black arrows. Interestingly, TQ-induces significant change in the pattern of motion in all four regions. The R1 motion is significantly organized in TQ-bound complex and shows a bending motion towards the TQ-binding site. In a ligand-free state, this region has a rather random pattern of motion. The R2 motion is simply changed from bending to twisting in the TQ-bound complex. The R3 region, which is composed of helix-loop-helix, changes its pattern of motion from twisting to bending in the TQ-bound complex. Although region R4 exhibits a bending motion, the direction of bending is apparently changed in the bound state. These essential dynamics play a role in protein function, and are reasonably constrained in the TQ-bound squalene monooxygenase complex and, thereby, TQ can potentially inhibit protein function.

Figure 6d displays the radius of gyration of the TQ-free (green curve) and bound (orange curve) squalene monooxygenase, which describes the compactness of the protein. A lower mean value of *g_(r)_* in TQ-bound squalene monooxygenase (26.53 ± 0.29 Å) indicates a more compact structure than the reference TQ-free protein, which further endorses the conclusions drawn from the RMSD, RMSF and PC1 analyses.

**Figure 8:**
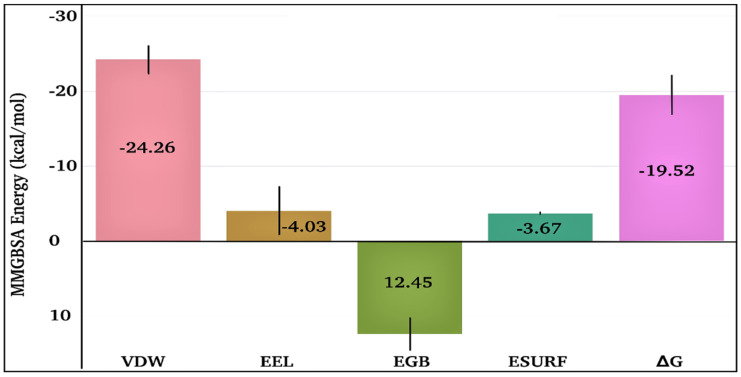
Individual energy components contribute to the total MMGBSA binding energy for the TQ-squalene monooxygenase complex. VDW, EEL, EGB, ESURF, and ΔG represent van der Waals, electrostatics, polar solvation, nonpolar solvation, and total MMGBSA binding energy, respectively.

The time-dependent MMGBSA binding energy for the TQ-squalene monooxygenase complex is displayed in Figure 6f; the individual energy components contributing to the total ΔG are given in Figure 8. The mean ΔG is observed to be −19.52 ± 2.62 kcal/mol, which is reasonably good for the ligand-protein complex. The major favorable energy contribution to the mean ΔG comes from van der Waal’s interactions (VDW), which is also supported by the molecular docking study. Electrostatic interactions (EEL) and nonpolar solvation (EGB) energy slightly favor the total binding energy. Being a semi-standard nonpolar molecule, the energy contribution from polar solvation (EGB) is small.

The frequent discontinuity in the formation of H-bonds between TQ and squalene monooxygenase (Figure 6e) further endorses the insignificant energy contribution from electrostatic interaction. The mean number of H-bonds is only 0.47 ± 0.63, which is in line with the higher VDW energy contribution to the total ΔG.

**Figure 9:**
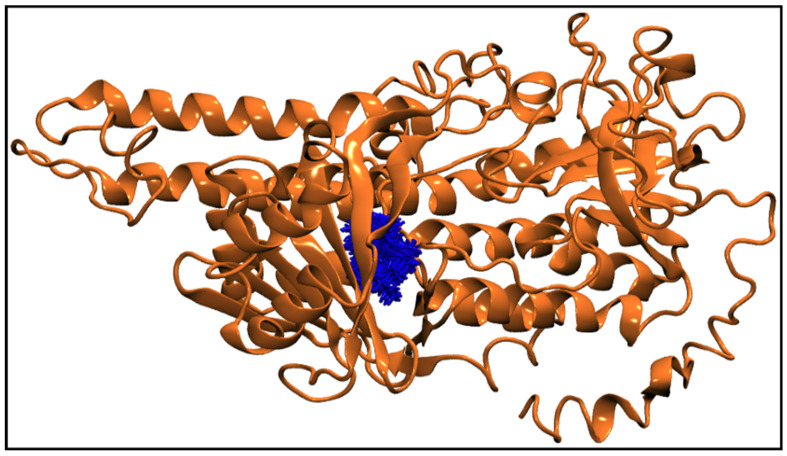
100 Snapshots of TQ (blue licorice representation) taken at every 1 ns from a 100 ns long trajectory showing the spatial distribution of the ligand in the protein binding site. The squalene monooxygenase protein (cartoon representation) is shown at 0 ns, representing the initial conformation of the protein.

Figure 6g displays the center-of-mass distance between TQ and squalene monooxygenase during MD simulation, which indicates that TQ remained in a bound form with the protein with a mean distance of 9.54 ± 0.69 Å. Figure 9 pictorially describes the spatial conformation of TQ in the binding site of squalene monooxygenase.

### 2.3. Statistical Analysis

There is a statistically significant difference (*p* < 0.05) between the mean values of the antipromastigote and antiamastigote activities of TQ; *t(4)* = −131.830, *p* = 0.000 (Table 4).

## 3. Discussion

TQ, a main pharmacologically active ingredient of *N. sativa*, exhibits substantial antiparasitic activity against several leishmanial species [20,21,22,23]; however, the antileishmanial activity of TQ against *L. major* in particular has not been reported. Moreover, there are no in silico studies of possible target proteins of TQ in any *Leishmania* species. Thus, our current study explored the in vitro and in silico antileishmanial potential of TQ against *L. major*, which fills the gap between antileishmanial activity and the mode of action of TQ against *L. major*.

This study demonstrates that TQ has significant antileishmanial activity against both the infectious stages, promastigotes and amastigotes of *L. major* (in comparison to control positive, untreated groups). The results further demonstrated that TQ is more toxic than CTL-D. The results also revealed that TQ is more potent against promastigotes than amastigotes of *L. major*.

Al-Turkmani et al. evaluated the antileishmanial activity of TQ against the promastigotes of *L. tropica* and found an IC_50_ (EC_50_) value of 7.92 μM, which supports our results demonstrating that the EC_50_ value of TQ is 2.62 ± 0.12 μM [24]. This comparison of data demonstrates that the activity in our study was higher than that in Al-Turkmani et al.

Abamor et al. reported that *N. sativa* essential oil encapsulated poly-ε-caprolactone (PCL) nanoparticles exhibit substantial antileishmanial activity against *L. infantum* promastigotes and amastigotes [20,21], which endorses our experimental findings that TQ, being an active ingredient of *N. sativa* essential oil, is active against promastigotes and amastigotes of *L. major*.

In another study, Islamuddin et al. found that TQ exhibits significant antileishmanial activity against promastigotes and amastigotes of *L. donovani* and demonstrated that the IC_50_ values for the promastigotes and amastigotes were 6.33 ± 1.21 μM and 7.83 ± 1.65 μM, respectively. These results further align with our results of IC_50_ values for the promastigotes and amastigotes of *L. major*, i.e., 2.62 ± 0.12 μM and 17.52 ± 0.15 μM, respectively [22]. This comparison of data reveals a higher antipromastigote activity of TQ in our study, but a higher antiamastigote activity of TQ in their study. This variation in the data is likely due to the different species of *Leishmania* used.

Furthermore, Mahmoudvand et al. demonstrated that TQ had significant antileishmanial activity against the promastigotes and amastigotes of *L. tropica* and *L. infantum*. The IC_50_ values for the antipromastigotes of *L. tropica* and *L. infantum* were 7.06 μM and 8.95 μM, respectively, while IC_50_ values for the antiamastigotes of *L. tropica* and *L. infantum* were 12.79 μM and 15.85 μM, respectively [23]. These results again support our results demonstrating that TQ has EC_50_ (IC_50_) values for the promastigotes and amastigotes of *L. major* of 2.62 ± 0.12 μM and 17.52 ± 0.15 μM, respectively. The antipromastigote activity of TQ in our study is higher than that of Mahmoudvand et al., but the antiamastigote activity is lower, again likely due to the different species of *Leishmania* used.

The comparison of our in vitro antileishmanial activity of TQ with previously published results reveals that TQ has generally higher antileishmanial activity against promastigotes and amastigotes of *L. major* species. This difference is likely due to the species difference and variability in lab conditions.

In addition, the present results show that promastigotes are more sensitive to TQ than amastigotes, which is consistent with many of our previous findings. This is because amastigotes are intracellular parasites that find protection inside the cells and have little exposure to the drugs, unlike the motile promastigotes, which are directly exposed to the drugs [25].

The molecular docking studies of TQ illustrated that squalene monooxygenase is the most preferred target protein for TQ. Furthermore, the MD simulation revealed that the TQ-squalene monooxygenase complex was highly stable during the entire simulation process conducted for 100 ns. Our literature search showed that this is the first-ever in silico study conducted for TQ against antileishmanial drug target proteins.

In conclusion, our findings reveal that TQ exhibits significant antiprotozoal (antileishmanial) activity against the promastigotes and amastigotes of *L. major*. Thus, TQ has great potential as an alternative antileishmanial drug candidate to treat drug-resistant leishmaniasis.

This work has some limitations, such as the fact that we only performed in vitro and in silico antileishmanial investigations of TQ; a post-in silico investigation is required to validate the in silico findings. However, we were unable to conduct these investigations at this time. Thus, before employing TQ as a therapeutic agent for drug-resistant leishmaniasis, further investigation, such as post-in-silico studies and human toxicity testing, are required.

## 4. Materials and Methods

### 4.1. Chemicals and Reagents

All of the required chemicals were procured from Sigma-Aldrich (Milwaukee, Brookfield, WI, USA) unless a specific company was recommended for some chemicals or accessories. TQ was procured from Sigma-Aldrich (Milwaukee, WI, USA) and had a purity of 99.0%. FT-IR was performed to validate the identity and purity of TQ [5].

### 4.2. Test Organism and Cultural Conditions

The test organism (*L. major*) was isolated from a Saudi national patient in promastigote form. Formal permission was obtained from the hospital to utilize this test organism for future research. The promastigotes of *L. major* initially obtained from a male patient were cultured weekly at 26 °C in sterile culture tubes in Schneider’s Drosophila medium (SDM) supplemented with 10% (*v*/*v*) heat-inactivated fetal bovine serum (FBS). Cryopreservation of 3 × 10^6^ parasites/mL was done in liquid nitrogen. The parasite’s virulence was maintained by injecting the promastigotes into the hindfoot of female BALB/c mice and isolating amastigotes after eight weeks while the mice were kept in a pathogen-free environment. The transformation of amastigotes to promastigotes was achieved by culturing again in 10% FBS supplemented SDM media at 26 °C. Transformed promastigotes that had been passaged less than five times were then used for infection and antiparasitic studies [2,3,4].

### 4.3. In Vitro Antileishmanial Activity of TQ

#### 4.3.1. Antipromastigotes Activity

Promastigotes were seeded onto 96-well microtiter plates at a density of 10^6^ cells/mL (200 µL/well) in Roswell Park Memorial Institute (RPMI)-1640 culture medium (phenol red-free) supplemented with 10% (*v*/*v*) FBS. The parasites were collected during their exponential growth stage and quantified using a hemocytometer. The antipromastigote activity of TQ and the standard drug was evaluated at various concentrations, including 25, 8.3, 2.7, 0.9, 0.3, and 0.1 µg/mL. Amphotericin B was used as a positive control, and dimethyl sulphoxide (DMSO) as a negative control. After 72 h of incubation, the tetrazolium dye assay (MTT) was performed to count live promastigotes and determine parasite growth inhibition. All the tests were performed in triplicate, and their readings were recorded at 540 nm using an ELISA reader, and the results were expressed as mean ± SD [2,3,4,5].

#### 4.3.2. Antiamastigote Activity

The activity of the test compounds against amastigotes were tested as per a previously published method [2,3,4,5]. Macrophages of peritoneal origin were isolated from Female BALB/c mice aged for six to eight weeks by peritoneal aspiration following thioglycolate stimulation for four days. Macrophages were cultured at a cell density of 5 × 10^4^ in microtiter plate wells with RPMI-1640 culture medium supplemented with 10% (*v*/*v*) FBS. The microtiter plates were then kept for 4 h in a humidified incubator at 37 °C containing 4% CO_2_ to encourage cell attachment. The attached macrophages were washed twice with PBS and then refreshed with 200 µL of RPMI-1640 medium (phenol-free, supplemented with 10% (*v*/*v*) FBS) containing promastigotes of *L. major* in a ratio of 10 promastigotes: 1 macrophage. The promastigotes were allowed to infect the macrophages for 24 h under optimum culture growth conditions. The free promastigotes were removed by washing with PBS three times, followed by the addition of TQ compound, a positive and negative control, and 200 µL of RPMI-1640 culture medium (phenol red-free, containing 10% (*v*/*v*) FBS). TQ and all controls were diluted serially to final concentrations of 25, 8.3, 2.7, 0.9, 0.3, and 0.1 µg/mL. After that, the microtiter plates were incubated for 72 h at 37 °C in the presence of 5% CO_2_. Amphotericin B was used as a positive control, and DMSO as a negative control with the same concentrations. The percentage of infection was determined after Giemsa staining. Each test was performed in triplicate, and results were expressed as mean ± SD [2,3,4,5].

#### 4.3.3. In Vitro Cytotoxicity Activity

To assess the cytotoxicity of TQ, the macrophages were subjected to an MTT cell viability assay. For this, the mice macrophages, maintained in RPMI-1640 medium (10% FBS) in 96 well culture plates with a cell density of 5000 cells per well, were treated with our test compound for 72 h at varying concentrations, i.e., 25, 8.3, 2.7, 0.9, 0.3, and 0.1 µg/mL. The cells with media only served as the negative control. The supernatant was then removed, and 50 μL of plain RPMI-1640 medium containing 0.5% *w*/*v* MTT was added and incubated for 4 h. Finally, 200 μL of DMSO was added to solubilize the insoluble MTT product. The xMark™ microplate absorbance spectrophotometer (Bio-Rad Laboratories, 1000 Alfred Nobel Drive, Hercules, CA, USA) was used for colorimetric analysis programmed for a 540 nm incidence wavelength. The microplate reader then read the absorbance at the given wavelength, and the readings were used to quantify cytotoxic effects in terms of CC_50_. Each test was performed in triplicate, and the results were expressed as mean ± SD [2,3,4,5].

### 4.4. In Silico Antileishmanial Activity of TQ

#### 4.4.1. Molecular Docking Studies

Sixteen reported enzyme receptors [5] were selected to dock with TQ (Table S1). The amino acid sequences of all proteins were obtained from the UniProt database. Based on the availability of the template proteins and their percentage identity, the 3D structures were either downloaded from the PDB and/or modeled from a Swiss model or I-tasser. Table S1 provides the FASTA sequences of all the proteins. Following validation of the structures, the .pdbqt files were created with AutoDock tools [26] using Kollman charges, respectively. A grid box in the x, y, and z dimensions was defined to include the entire protein to execute blind docking. Grid spacing was fixed to 1 Å following the AutoDock vina program’s recommendations [27]. TQ’s three-dimensional structure was retrieved from the PubChem database (CID 10281) [28]. The Gasteiger–Marsili empirical atomic partial charges (determined based on electronegativity equilibration) [29] were stabilized for TQ (ligand), while the pdbqt file was created using a Raccoon graphical interface [30]. The last step was running the molecular dockings using the AutoDock vina tool [27] using an exhaustiveness value of 80. For analysis, 20 docked conformations were produced for each system.

#### 4.4.2. Molecular Dynamics (MD) Simulations

The squalene monooxygenase protein [5] in free-form and in complexed form with TQ were subjected to a molecular dynamics simulations study. The protonation states were evaluated at pH 7.4 using the PlayMolecule ProteinPrepare web server (https://playmolecule.com/; accessed on 1 February 2022) for His, Lys, Arg, Asp, and Glu residues and implemented after visual inspection [31]. Both ligand-free and TQ-bound squalene monooxygenase systems were solvated with a TIP3P water model in a truncated octahedral box. The lowest distance between protein systems and the simulation box’s edges was set at 10 Å to fulfill the minimum image convention criteria efficiently. The KCl ionic concentration in both systems was set to 0.15 M by adding 87 K^+^ ions and 104 Cl^−^ ions to the environment. The ligand-free and TQ-bound squalene monooxygenase systems contained 101,654 and 101,555 atoms, respectively. The Chemistry at HARvard Macromolecular Mechanics Graphical User Interface (CHARMM-GUI) webserver was utilized to produce all input data files [32,33].

For 5000 steps, both systems were minimized using the steepest descent approach, and convergence was attained under the force limit of 1000 (kJ/mol/nm) to relax any steric clashes followed by equilibration at NVT (canonical ensemble: where moles, N; volume, V; and temperature, T were conserved) and NPT (isothermal-isobaric ensemble: where moles, N; pressure, P; and temperature, T were conserved) ensembles for 100 ps (50,000 steps) and 1000 ps (1,000,000 steps), respectively, using time steps 0.2 fs, at 310.15 K to ensure a fully converged system for the production run [5].

The simulation runs for both systems were conducted at a constant temperature of 310.15 K and a pressure of 1 atm or 1 bar (using an NPT ensemble), utilizing weak coupling velocity rescaling (modified Berendsen thermostat) and the Parrinello-Rahman algorithm, respectively. The relaxation times were set at τ, T = 0.1 ps and τ, *p* = 2.0 ps. Using the LINear Constraint Solver (LINCS) algorithm, all bond lengths involving hydrogen atoms were maintained at the optimal bond lengths, with a time step of 2 fs. Newton’s equation of motion was integrated using the Verlet algorithm. The forces and key electrostatic interactions beyond the short-range cutoff (>10 Å) were determined by implementing the particle mesh Ewald (PME) method. Periodic boundary conditions (PBCs) were employed in all directions of x, y, and z. For each of the complexes, the production was run for 100 ns. The GROMACS simulation package (GROMACS 2020.4) [34] was utilized to execute molecular dynamics simulations (MDS) using the all-atom additive CHARMM36 m forcefield for protein [35], and CHARMM general force field (CGenFF) [36,37] was implemented for all four ligands. GRaphing, Advanced Computation, and Exploration of data (Grace) was used to generate all plots (https://plasma-gate.weizmann.ac.il/Grace; accessed on 1 February 2022). The molecular mechanics Generalize Born surface area (MMGBSA) receptor-ligand binding energies were computed at every 1 ns of simulation for all four complexed systems [38]. The Gibbs binding free energy was calculated using the single trajectory approach using the following equation, as discussed in detail by Genheden & Ryde [39].

(1) $$\Delta G_{binding}=G_{complex}-G_{protein}-G_{ligand}$$

where G can be calculated as:

(2) $$G=E_{ele}+E_{vdW}+G_{pol}+G_{np}-TS$$

where $E_{ele}\;\mathrm{and}\;E_{vdW}$ are standard MM energy terms representing electrostatic and van der Waals interactions. Since the bounded terms are canceled out in the single trajectory approach, they are not mentioned here. $G_{pol}\;\mathrm{and}\;G_{np}$ are the polar and nonpolar contributions to the solvation free energy, calculated by solving the Generalized Born equation and obtained from a linear relation to the solvent accessible surface area (SASA), respectively. *TS* is the configurational entropy, which calculates the number of available configurations occupied by a molecule in 3D space multiplied by the temperature, and is often neglected due to the increased computational cost associated with it. The Gibbs free energy calculation method is widely recognized and used in several studies [5,16].

### 4.5. Statistical Analysis

The linear regression equation for the EC_50_ and CC_50_ values were calculated using Microsoft Excel. The SI values were derived by dividing the CC_50_ of each parasite by the EC_50_. The significance of the differences between the mean values of antipromastigote and antiamastigote activities of TQ were determined using an unpaired independent samples *t*-test with a significance level of *p* = 0.05. The statistical analyses were conducted using SPSS software, version 20.0 (IBM, Armonk, NY, USA) [5].

## Figures and Tables

**Table 1:** Antipromastigote activity of TQ and CTL-D.

Compounds	Antipromastigote Activity		
	EC_50_ (μM)	CC_50_ (μM)	SI
TQ	2.62 ± 0.12	29.54 ± 0.07	11.27
CTL-D	0.84 ± 0.04	8.01 ± 0.29	9.54

**Table 2:** Antiamastigote activity of TQ and CTL-D.

Compounds	Antiamastigote Activity		
	EC_50_ (μM)	CC_50_ (μM)	SI
TQ	17.52 ± 0.15	29.54 ± 0.07	1.69
CTL-D	0.50 ± 0.06	8.01 ± 0.29	16.02

Note: All the tests were conducted in triplicate, and the results are presented as mean ± SD.

**Table 3:** Protein-ligand predicted binding energy obtained from molecular docking studies.

#	Enzymes	Pathway	Binding Energy (kcal/mol)
1.	Squalene monooxygenase	Sterol biogenetic pathway	−7.1
2.	Fructose-bisphosphate aldolase	Glycolytic pathway	−6.8
3.	Mannosyltransferase (GPI-14)	Glycosylphosphatidylinositol-anchor biosynthesis	−6.8
4.	Trypanothione reductase	Trypanothione pathway	−6.6
5.	Trypanothione synthetase-amidase	Trypanothione pathway	−6.4
6.	Phosphoglycerate kinase	Glycolytic pathway	−6.3
7.	Deoxyhypusine hydroxylase	Hypusine biosynthetic	−6.3
8.	Adenine phosphoribosyltransferase	Purine salvage pathway	−6.2
9.	Xanthine phosphoribosyltransferase	Purine salvage pathway	−6.2
10.	Squalene synthase	Sterol biogenetic pathway	−6.1
11.	Farnesyl pyrophosphate synthase	Sterol biogenetic pathway	−5.9
12.	Pyruvate kinase	Glycolytic pathway	−5.9
13.	Tryparedoxin peroxidase	Trypanothione pathway	−5.8
14.	Phosphoglycerate mutase (2,3-diphosphoglycerate-independent)	Glycolytic pathway	−5.8
15.	Glyceraldehyde-3-phosphate dehydrogenase	Glycolytic pathway	−5.6
16.	Triosephosphate isomerase	Glycolytic pathway	−5.1

**Table 4:** Statistical analysis of antipromastigote and antiamastigote activities of TQ.

Independent Samples Test										
		Levene’s Test for Equality of Variances		*t*-Test for Equality of Means						
		F	Sig.	*t*	df	Sig. (2-Tailed)	Mean Difference	Std. Error Difference	95% Confidence Interval of the Difference	
									Lower	Upper
EC_50_	Equal variances assumed	0.203	0.676	−131.830	4	0.000	−2.44667	0.01856	−2.49820	−2.39514
	Equal variances not assumed			−131.830	3.806	0.000	−2.44667	0.01856	−2.49925	−2.39409

## Data Availability

Not applicable.

## Supplementary Materials

The following supporting information can be downloaded at: https://www.mdpi.com/article/10.3390/antibiotics11091206/s1, Figure S1: FT-IR spectra of thymoquinone (TQ); Figure S2: FT-IR spectra of standard TQ; Figure S3: Snapshots of TQ-free (green) and bound states (orange) squalene monooxygenase protein at various timescales; Table S1: The selected proteins for molecular docking studies with TQ ligand targeting multiple pathways in *Leishmania* spp. The table also lists the name of enzymes, their FASTA sequences, amino acid lengths, their PDB codes (if existed) or models (generated from homology modeling approach or threading approach), and the corresponding pathways where the protein is involved.
