# Supplementary material for: Antiprotozoal Activity of Thymoquinone (2-Isopropyl-5-methyl-1,4-benzoquinone) for the Treatment of Leishmania major-Induced Leishmaniasis: In Silico and In Vitro Studies
Source: Antibiotics (Basel). 2022 Sep 6;11(9):1206. doi: 10.3390/antibiotics11091206 (PMC9495120; doi:10.3390/antibiotics11091206)
Supplement: Supplementary file 1 [file antibiotics-11-01206-s001.zip › antibiotics-1748664-supplementary.pdf]

Supplementary Material

Antiprotozoal Activity of Thymoquinone (2-Isopropyl-5-methyl-1,4-benzoquinone) for the Treatment of *Leishmania major*-Induced Leishmaniasis: In Silico and In Vitro Studies

Kamal A. Qureshi[1,*] Mahrukh Imtiaz[2], Ibrahim Al Nasr[3,4], Waleed S. Koko[3], Tariq A. Khan[5], Mariusz Jaremko[6], Syed Mahmood[7], M. Qaiser Fatmi[2,*]

[1] Department of Pharmaceutics, Unaizah College of Pharmacy, Qassim University, Unaizah 51911, Saudi Arabia
[2] Department of Biosciences, COMSATS University Islamabad, Islamabad 45600, Pakistan; mahukhimtiaz92@yahoo.com (M.I.)
[3] Department of Laboratory Sciences, College of Science and Arts, Qassim University, Ar Rass 51921, Saudi Arabia; insar@qu.edu.sa (I.A.N.); wa.mohamed@qu.edu.sa (W.S.K.)
[4] Department of Biology, College of Science and Arts, Qassim University, Unaizah 51911, Saudi Arabia
[5] Department of Clinical Nutrition, College of Applied Health Sciences, Qassim University, Ar Rass 51921, Saudi Arabia; tara.khan@qu.edu.sa (T.A.K.)
[6] Smart-Health Initiative (SHI) and Red Sea Research Center (RSRC), Division of Biological and Environmental Sciences and Engineering (BESE), King Abdullah University of Science and Technology (KAUST), Thuwal 23955, Saudi Arabia; mariusz.jaremko@kaust.edu.sa (M.J.)
[7] Department of Pharmaceutical Technology, Faculty of Pharmacy, Universiti Malaya, Kuala Lumpur 50603, Malaysia; syedmahmood@um.edu.my (S.M.)
* Correspondence: ka.qurishe@qu.edu.sa (K.A.Q.); qaiser.fatmi@comsats.edu.pk (M.Q.F.)

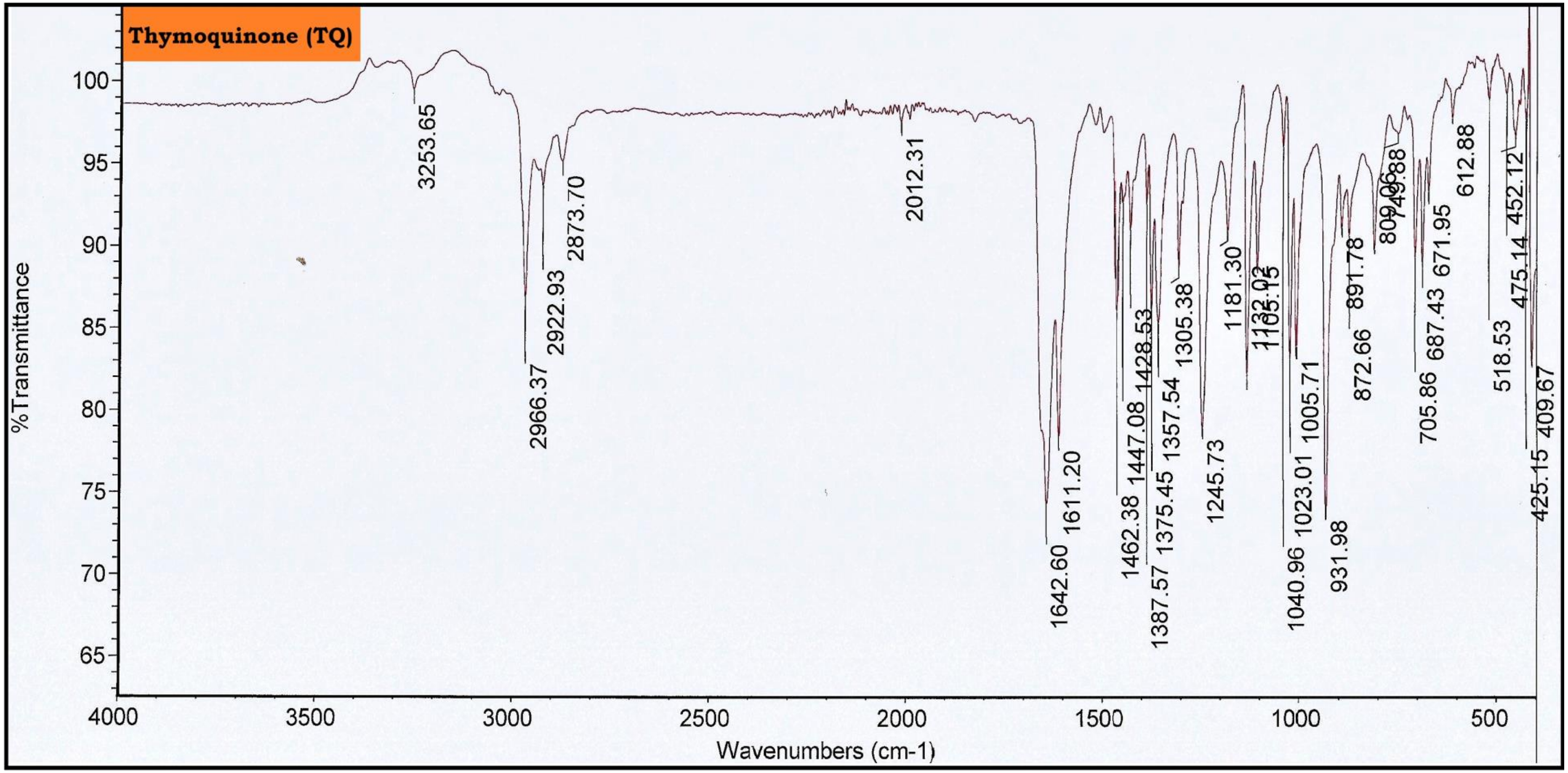

Figure S1. FT-IR spectra of thymoquinone (TQ).

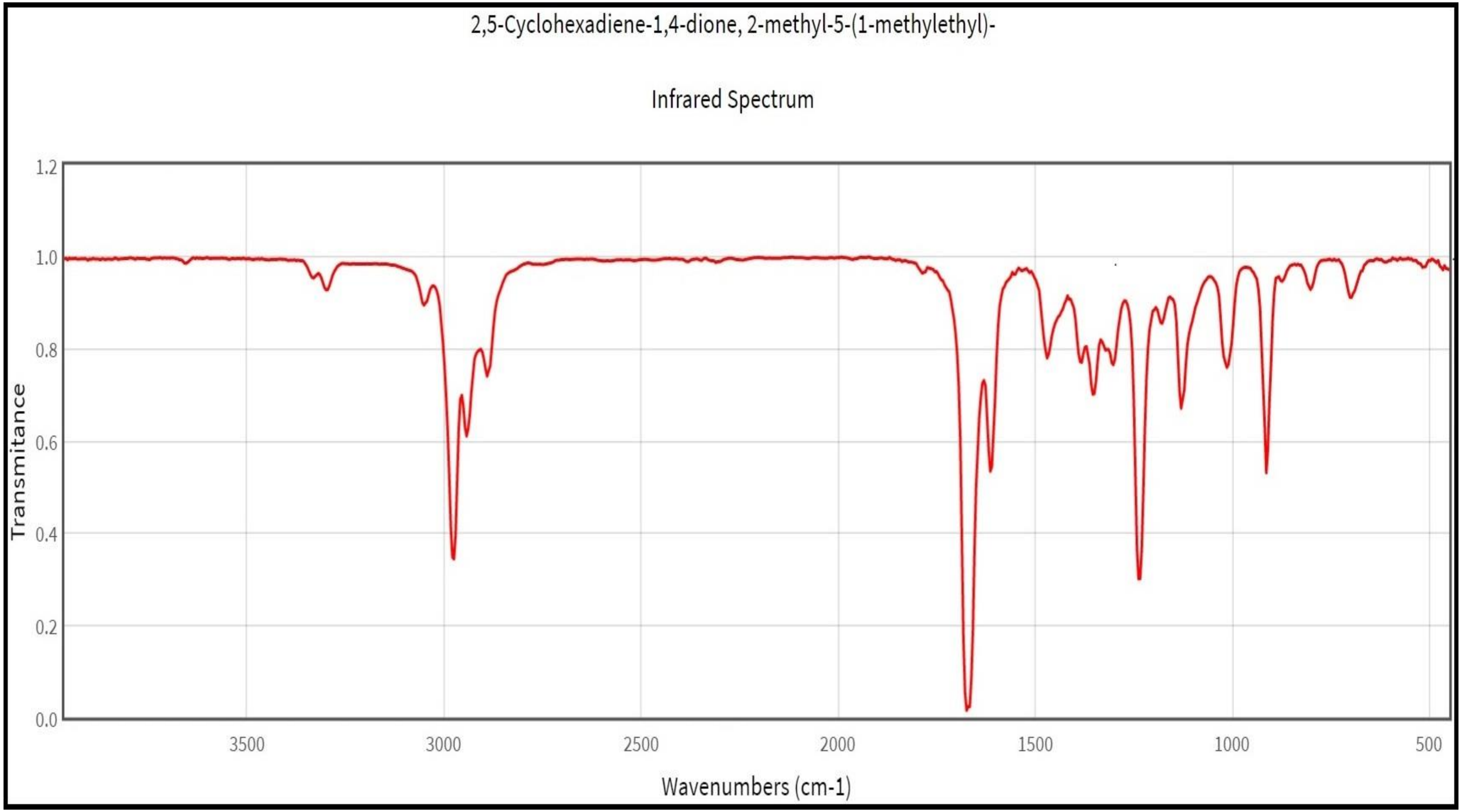

Figure S2. FT-IR spectra of standard TQ. (Source : https://webbook.nist.gov/cgi/cbook.cgi?ID=C490915&Mask=80)

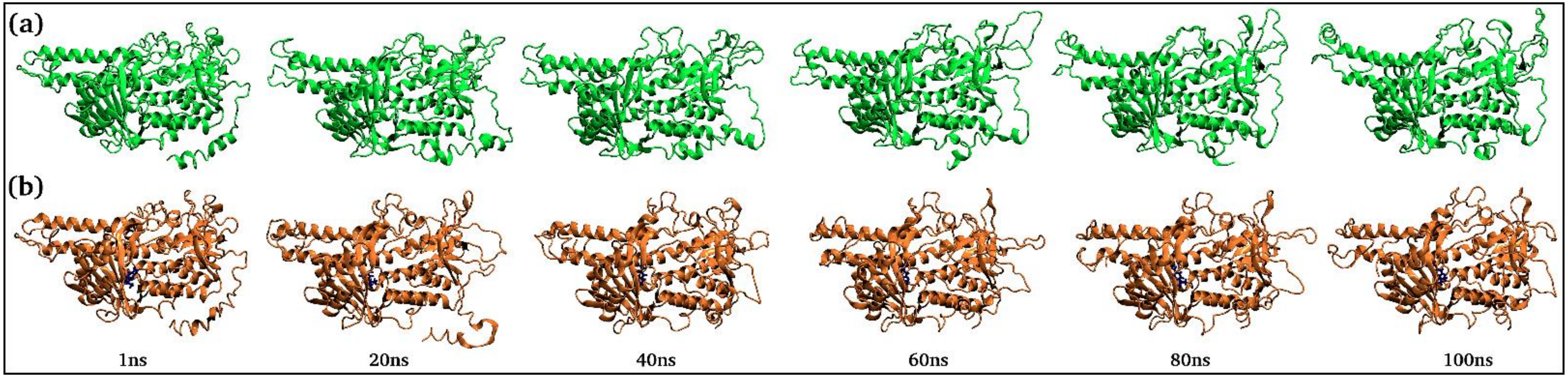

Figure S3. Snapshots of TQ-free (green) and bound states (orange) squalene monooxygenase protein at six different timescales, i.e., 1 ns, 20 ns, 40ns, 60 ns, 80 ns and 100 ns, during MD simulation study, indicating various conformational changes in the protein. As indicated, TQ remains in bound form throughout the simulation.

Table S1. The selected proteins for molecular docking studies with TQ ligand targeting multiple pathways in *Leishmania spp. The table also lists the name of enzymes, their FASTA sequences, amino acid lengths, their PDB codes (if existed) or models (generated from homology modeling approach or threading approach), and the corresponding pathways where the protein is involved.*

#	Enzyme	Organism	FASTA	Amino acid	PDB/Model	Pathway
1.	Trypanothione reductase	*L. infantum*	MGSSHHHHHHSSGLVPRGSHMS<br>RAYDLVVLGAGSGGLEAGWNA<br>AVTHKKKVAVVDVQATHGPPLF<br>AALGGTCVNVGCVPKKLMVTGA<br>QYMDLIRESGGFGWEMDRESLCP<br>NWKTLIAAKNKVVNSINESYKSM<br>FADTEGLSFHMGFGALQDAHTV<br>VVRKSEDPHSDVLETLDTEYILIAT<br>GSWPTRLGVPGDEFCITSNEAFYL<br>EDAPKRMLCVGGGYIAVEFAGIF<br>NGYKPCGGYVDLCYRGDLILRGF<br>DTEVRKSLTKQLGANGIRVRTNL<br>NPTKITKNEDGSNHVHFNDGTEE<br>DYDQVMLAIGRVPRSQALQLDK<br>AGVRTGKNGAVQVDAYSKTSVD<br>NIYAIGDVTNRVMLTPVAINEGA<br>AFVETVFGGKPRATDHTKVACA<br>VFSIPPIGTCGMTEEEAAKNYETV<br>AVYASSFTPLMHNISGSKHKEFMI<br>RIITNESNGEVLGVHMLGDSAPEI<br>IQSVGICMKMGAKISDFHSTIGVH<br>PTSAEELCSMRTPAYFYESGKRVE<br>KLSSNL	511	2JK6	Trypanothione pathway

2.	Mannosyltransferase (GPI-14)	*L. braziliensis*	MRNKTWLDRQSIGTLLLYGGLAR VVLLIYAAFHDYYFRVKYTDIDY MIVVDGARELLHGGTPFDRTTYR YTPLLAVLVIPAVLIANPLGKVVF ALSDLGAAYYCFHMLLRFSTERS AKWMVVILILFNPVVLNVSTRGN SDMLISCMSMGVLAKFAEGRYFT AASILGFAVHFKIYPIIYALPLVLG VWERAKQERFFGRLAHTASVVV GCGLCFTVAFAVPTYVCYLVYGQ QYLDEGFIYHIHREDHRHNFSPY WLLMYLNMGRRDLGVGVDYSA GLFAFLPQFAVLCYASWKLRKNI AHACCVETILFVAFNKVCTVQYF VWFLPFLAFVFCEPAQPKWLTGV TNAPKSERPSMLSAVAAILMWSL MIPLWVWTAYGLEFEGRNHYGR LWIVSCVFYLATVSLAAWLGRLC YRSRITVSIKDHRPASKRA	432	I-Tasser (Various templates were used including: 5ezm.pdb, 5f15.pdb, 6snh.pdb)	Glycosylphosphatidylinositol-anchor biosynthesis
3.	Trypanothione synthetase-amidase	*L. major*	MSSLQRASVSFNKPGHIPFGAVQ GYAPGGVPAYSNKHDHYFSGER NIEDNIFFGFKYQCVEFARRWLLV RKGLLLPDVNWACHIFQLKEVR DAATTESFAVLQVRNGTTTKPEA DALLVYPSTDANPVGHVGTITEV GDDYVCVADQNYRFHKWESSCA YKLKLDHRDGIWTIIDDIDADEIEI PLGWLTFPGRANRPEGAPPVALH	652	2VOB	Trypanothione pathway

			PSLHFKEPPKPYLLRRNFLPTESK<br>ANWLDMNNPAERLFVEEFGMD<br>VSRTRLEEKVVSYYESNHEFHLRC<br>VAYGTQLHAIFMEATAQVIESDE<br>KLRLFAIPEEFWPRIRHSWKYQQT<br>YISGRFDFAFNNETGEVKCFEYN<br>ADSASTLLECGLIQQKWAESVGL<br>DKQDTRGSGFAVERNLKMAWA<br>NSGATGRVHFCVDEEREEQYTAL<br>YCMQAAEAVGLEGKLCILFDEFR<br>FDDNGHVVDSDGVRVRNVWKT<br>WMWESAITDYYAAREERGENWK<br>PSPKDKVRLCDLLLGDDWEILYFE<br>PMWKVIPSNKAILPMIYHNHPEH<br>PAILKAEYELTDELRKHGYAKKPI<br>VGRVGSNVIITSGDGVVHAESGG<br>KYGKRNMIYQQLFELKKQDDYY<br>AIIGGWMIGDAFSGTGIREDKSVI<br>TGVDSPFAAVRIKTDKLPHPVTLK<br>DIDKMAEDE			
4.	Tryparedoxin peroxidase	*L. major*	MSCGNAKINSPAPSFEEVALMPN<br>GSFKKISLSSYKGKWVVLFFYPLD<br>FTFVCPTEVIAFSDSVSRFNELNCE<br>VLACSIDSEYAHLQWTLQDRKKG<br>GLGTMAIPMLADKTKSIARSYGV<br>LEESQGVAYRGLFIIDPHGMLRQI<br>TVNDMPVGRSVEEVLRLLEAFQF	199	**4K1F**	Trypanothione pathway

			VEKHGEVCPANWKKGAPTMKPE<br>PNASVEGYFSKQ			
**<u>5.</u>**	**<u>Squalene synthase</u>**	*L. major*	MGFFSDSVAMMRVKWQMRSVKI<br>QVPPEETDLRFCYDIMNDVSRSFA<br>VVVAQLADQQLRDAICIFYLVLR<br>ALDTLEDDMSVPVDVKLKELPKF<br>HTHTSDMSWCMSGVGEGREREL<br>LAKYPCVSREFKKLKKEYQDVIA<br>NICERMANGMCEFLKRPVVTKD<br>DYNQYCHYVAGLVGHGLTQLFA<br>RCGFEDPSLDDDLTSSNHMGLFL<br>QKTNIIRDYYEDIREEPPRMFWPK<br>EIWGTYVTELKELKSESNNAAAV<br>QCLNAMVADALVHVPYIVDYLS<br>ALRDPSVFRFCAIPQVMAIATLKE<br>VYNNPDTFQVKVKVSRPESCRIM<br>LKATTLYSSLSMFRDYCVELQEKL<br>DMQDASSVSIANSLAAAIERIDLQ<br>LKKCQDVSYTRSLLARYPGLGGQ<br>FLLTVMDTVAGFFGGRKEIAGHA	414	Swiss Model (**Template used:** 3wca.pdb **Query Coverage:** 82% **Sequence Identity:** 61.83%)	Sterol biosynthetic pathway
6.	Squalene monooxygenase	*L. major*	MLYFFAAVLCAVSTLLLLNRTLSR<br>LRLSPARTSYDYDVIIVGGSIAGPV<br>LAKALSDQGRKVLMVERTLFTKP<br>DRIVGELLQPGGLNALKEVGMKE<br>CAETIGMPCHGYVVVDHKGKQV<br>DLPYRKGASGVSFHFGDFVQSLRS<br>HVFHNCKANVTMVEGTVNNILT<br>EGLSFSERAYGVEYTIAEKYEVPT	569	I-Tasser (Various templates were used including: 6c6n.pdb, 6fho.pdb, 6c6r.pdb)	Sterol biosynthetic pathway

			KPFREDPPKTNPVAATVRKVATA<br>PLVVMCDGGMSKWKSRYQHYTP<br>AYEYHSHFIGLVLKTVRLPKEQRG<br>TVFFGKTGPILSYRLDDNELRLLV<br>DYNKPTLPSLEKQSEWLIQDVAP<br>CLPENMREQFVRVSKDTKSLRSM<br>PMARYPPAFPSIKGYVGIGDHAN<br>QRHPLTGGGMTCCFRDAIRLANS<br>LNGIQSLRSVNQEEMAAIEDKIQ<br>AAILNYARYRYTHSCCINLLSWA<br>LYSVFSSPALRDACFDYFLCGGNC<br>VTGPMDLLAGLDPNVGSLFFHYC<br>CVMLHGVANVMMRTGAYSESG<br>KQLSNLEKLTNVASFFVDWERM<br>KHAAYLLGKSTQIALPLAKSEFYS<br>MWRFVDPTSPLANISKRIKTMVY<br>TKQFNGKQRKPVGL			
7.	Farnesyl pyrophosphate synthase	*L. major*	MAHMERFQKVYEEVQEFLLGDA<br>EKRFEMDVHRKGYLKSMMDTTC<br>LGGKYNRGLCVVDVAEAMAKD<br>TQMDAAAMERVLHDACVCGW<br>MIEMLQAHFLVEDDIMDHSKTRR<br>GKPCWYLHPGVTAQVAINDGLIL<br>LAWATQMALHYFADRPFLAEVL<br>RVFHDVDLTTTIGQLYDVTSMVD<br>SAKLDAKVAHANTTDYVEYTPF<br>NHRRIVVYKTAYYTYWLPLVMG<br>LLVSGTLEKVDKKATHKVAMVM	362	4JZX	Sterol biosynthetic pathway

			GEYFQVQDDVMDCFTPPEKLGKI<br>GTDIEDAKCSWLAVTFLTTAPAE<br>KVAEFKANYGSTDPAAVAVIKQL<br>YTEQNLLARFEEYEKAVVAEVEQ<br>LIAALEAQNAAFAASVKVLWSKT<br>YKRQK			
8.	Glyceraldehyde-3-phosphate dehydrogenase	*L. major*	MAPIKVGINGFGRIGRMVLQAIC<br>DQGLIGNEIDVVAVVDMSTNAEY<br>FAYQMKYDTVHGRPKYTVEAVK<br>STPSVKTPDVLVVNGHRIKCVKA<br>QRNPADLPWGKLGVDYVIESTGL<br>FTDKLQAEGHIKGGAKKVVISAP<br>ASGGAKTIVMGVNQHEYSPTSH<br>HVVSNASCTTNCLAPIVHVLTKE<br>NFGIETGLMTTIHSYTATQKTVDG<br>VSLKDWRGGRAAAINIIPSTTGA<br>AKAVGMVIPSTKGKLTGMSFRVP<br>TPDVSVVDLTFRSTRETSIQEIDKA<br>IKKAAQTYMKDILGFTNDELVSS<br>DFINDNRSSVYDSKATLQNNLPG<br>EKRFFKIVSWYDNEWGYSHRVVD<br>LVRYMAATDAASAKM	361	Swiss Model (**Template used:** 1gyq.pdb **Query Coverage:** 99% **Sequence Identity:** 94.69%)	Glycolytic pathway
9.	Triosephosphate isomerase	*L. major*	MSAKPQPIAAANWKCNGTTASIE<br>KLVQVLNEHNISHDVQCVVAPTF<br>VHIPLVQAKLRNPKYVVSAENAI<br>AKSGAFTGEVSMPILKDLGINWVI<br>LGHSERRTYYGETDETVAQKVAD<br>ACKQGFMVIACIGETLQQREANQ	251	Swiss Model (**Template used:** 5cg7.pdb	Glycolytic pathway

			TAKVVLSQTSAIATKLPKEAWDQ<br>IVLAYEPVWAIGTGKVATPEQAQ<br>EVHALLRKWVSEKIGTDVAAKLR<br>ILYGGSVSAGNAKTLYMKPDING<br>FLVGGASLKPEFRDIIDATR		**Query Coverage:** 100% **Sequence Identity:** 91.63%)	
10.	Phosphoglycerate kinase	*L. major*	MSLVLKKSIDDATVRDKKVLIRV<br>DFNVPVKNGKITNDFRIRSALPTI<br>QKVLKEGGSCILMSHLGRPKGAR<br>MSDPSPEKGVRGYEEAATLRPVA<br>ARIAELLGQKVEFAPDCLDAAAY<br>ASKLKNGDVLLLENVRFYAEEGS<br>KKEEERDAMAKVLASYADLYVS<br>DAFGTAHRDSATMTGIPKVLGA<br>GYAGYLMEKEINYFSRVLNNPPR<br>PLVAIVGGAKVSDKIELLDNMLS<br>RINYLVIGGAMAYTFQKAQGRKI<br>GISMCEEDKLDLAKSLLKKAQER<br>GVQVFLPVDHVCNKEFKAADSP<br>LVTESVDVPDGYMALDIGPRTIH<br>MYEEVIGRCKSAIWNGPMGVFE<br>MPCYSKGTFAVAKAMGTGTQKN<br>GLLSIIGGGDSASAAELSGEAKN<br>MSHVSTGGGASLELLEGKTLPGV<br>AILTDKDVKERGASCRFAFGVGS<br>PSREACPLRCGHIFGGASIVREIVK<br>LVVALLIGIFIGRRMSTKLIR	479	Swiss Model (**Template used:** 16pk.pdb **Query Coverage:** 86% **Sequence Identity:** 73.39%)	Glycolytic pathway

11.	Pyruvate kinase	*L. major*	MSQLAHNLTLSIFEPVANHRATRI VCTIGPSTQSVEALKGLIQSGMSV ARMNFSHGSHEYHQTTINNVRQ AAAELGVNIAIALDTKGPEIRTGQ FVGGEAVMERGATCYVTTDPAF ADKGTKDKFYIDYQNLSKVVRPG SYIYIDDGILILHVQSHEDEQTLKC TVTNAHTISDRRGVNLPGCDVDL PAVSAKDCADLQFGVEQGVDMI FASFIRSAEQVGDVRKALGAKGH DIMIICKIENHQGVQNIDSIIEESD GIMVARGDLGVEIPAEKVVVAQK ILISKCNVAGKPVICATQMLESMT YNPRPTRAEVSDVANAVFNGAD CVMLSGETAKGKYPNEVVQYMA RICLEAQSAVNEYVFFNSIKKLQPI PMSAEEAVCSSAVNSVYETKAKV MVVLSNTGRSARLVAKYRPNCPI VCVTTRLQTCRQLNITQGVESVFF DAEKLGHDEGKEQRVAMGVGF AKSKGYVQTGDYSVVIHADHKV KGYANQTRILLVE	499	Swiss Model (**Template used:** 3hqn.pdb **Query Coverage:** 100% **Sequence Identity:** 95.59%)	Glycolytic pathway
12.	Phosphoglycerate mutase (2,3-diphosphoglycerate-independent)	*L. major*	MSNLLLRPHKDLPRRKLLIVVMD GLGIGPEDEYDAVHMASTPFMD AQRQNSRHFRSVRAHGTAVGLP TDADMGNSEVGHNALGAGRVA LQGASLVDDALKSGEIYTGEGYR YLLGAFTKEGSTLHLIGLLSDGGV	553	Swiss Model (**Template used:** 3igy.pdb	Glycolytic pathway

			HSRDNQIHSIIEHAAKNGAKGIR VHVLYDGRDVPDGSSFRFTEELE AVLARVRQDGCDAAIASGGGRM FVTMDRYDADWSIVERGWKAQV LGDARHFHSAKEAITTFREEDPK VTDQYYPPFVVVDEQGKPLGTIE DGDAVLCVNFRGDRVIEMTRAFE DEDFDKFDRVRVPKVRYAGMMR YDGDLGIPNNFLVPPPKLTRVSEE YLCGTGLQIFACSETQKFGHVTYF WNGNRSGKVDEEHETFKEVPSD RVHFNEQPKMKSAEITEAAIEAL KSGMYDVVRINFPNGDMVGHTG DLRATIAGVEAVDESLAKLKDAV DSINGVFIVTADHGNSDDMAQR DKKGKPMKDEKGNVLPLTSHTLS PVPVFIGGAGLDPRVAMRTDLPT AGLANVTATFINLLGFEAPEDYEP SLIFVEN		**Query Coverage:** 100% **Sequence Identity:** 92.21%)	
13.	Fructose-bisphosphate aldolase	*L. major*	MSRVTIFQSQLPACNRIKTPYESEL IATVKKLTTPGKGLLAADESIGSC TKRFEPIGLSNTEEHRRQYRALML EAEGLEQYISGVILHDETVSQKAS NGKTFPEYLTARGVVPGIKTDMG LCPLLEGAEGEQMTEGLDGYVKR ASVYYKKGCRFCKWRNVYKIQN GTVSEPAVRFNAETLARYAILSQ MSGLVPIVEPEVMIDGKHDIDTC	371	Swiss Model (**Template used:** 2qap.pdb **Query Coverage:** 100%	Glycolytic pathway

			QRVSEHVWREVVAALQRHGVIW<br>EGCLLKPNMVVPGAESGQTAAP<br>EQVAHYTVMTLARTMPAMLPGV<br>MFLSGGLSEVQASEYLNAINNSPL<br>PRPYFLSFSYARALQSSALKAWG<br>GKDSGVAAGRRAFLHRARMNS<br>MAQLGKYKRADDDASSSSLYVK<br>GNIY		**Sequence Identity:** 95.96%)	
14.	Adenine phosphoribosyltransferase	*L. major*	MSFKEISPNSFLLEDSHPLSQLLKK<br>NYCWYSPVFSPRNVPRFADVSSIT<br>ESPETLKAIRDFLVQRYRTMSPAP<br>THILGFDARGFLFGPMIAVELGIP<br>FVLMRKADKNAGLLIRSEPYEKE<br>YKEAAPEVMTIRYGSISKGSRVVLI<br>DDVLATGGTALSGLQLVEASDA<br>MVVEMVSILCIPFLKAAEKIHSTG<br>HSRYKDIKFISLLSEEALTEDNCG<br>DSKNYTGPRVVSCGDVLSKHSQ	237	Swiss Model (**Template used:** 1qb7.pdb **Query Coverage:** 98% **Sequence Identity:** 92.27%)	Purine salvage pathway
15.	Xanthine phosphoribosyltransferase	*L. major*	MLPNHSCKGFVDAQGRVFVDGR<br>EYPMASGIVATEDVIQANIKAMA<br>NTIAKDYKSLSHRDVRLSPGTAA<br>TAEAAEAPISYDNPLIIISVLKGSYI<br>FTSDFIRYLGDCGLPHVVDFVRLA<br>SYNSDTTSTGQISMLAGLRFENLR<br>GKHVLIVEDVCDSGRTLRFLRDYI<br>MEKFQPKSVKTLVMVNKEQAAR<br>KLDFDPEYFCLAGPNKYIVGYGFE	238	Swiss Model (**Template used:** 6mxc.pdb **Query Coverage:** 92% **Sequence Identity:** 56.36%)	Purine salvage pathway

			VNDRYRDLRHIFILRDGEATRYPA<br>KL			
16.	Deoxyhypusine hydroxylase	*L. major*	MSASNSCTVEEVRKEYAKLLDPQ<br>EPLDSRMRELYRLKEDCLKTVAG<br>VTVILEAIDTTDSVLLQHELAYNA<br>GQSGREEAVPELERILRTTSYDVV<br>TRHEAAEALGAIGSPLALQVLET<br>HSAPTTEPEASIRETCELALARIA<br>MKETKGDAAVAPPSGCEFVSVDP<br>SPAFSALYSSTDEPVPLTVEELEAV<br>LLDTSGRTRLFRRYMAMFTLRNL<br>ATEAAVAALCRGLREDTISALFR<br>HEVAFVLGQLERPSSQPALIAALK<br>DEEEAPMVRHEAAEALGAIADP<br>ATLPVLESYATHHEPIVRDSCVVA<br>LEMHKYWAHFNSLAHQQQEA	325	I-Tasser (Various templates were used including: 4d4z.pdb, 6fsq.pdb)	Hypusine biosynthetic pathway
